# Depolarization-Induced Calcium-Independent Synaptic Vesicle Exo- and Endocytosis at Frog Motor Nerve Terminals

**Published:** 2013

**Authors:** M.M. Abdrakhmanov, A.M. Petrov, P.N. Grigoryev, A.L. Zefirov

**Affiliations:** Kazan State Medical University, Butlerov str., 49, Kazan, Russia, 420012

**Keywords:** motor nerve terminals, exocytosis, endocytosis, calcium, constant depolarization current, cadmium

## Abstract

The transmitter release and synaptic vesicle exo- and endocytosis induced by constant current depolarization of nerve terminals were studied by microelectode extracellular recording of miniature endplate currents and fluorescent microscopy (FM 1-43 styryl dye). Depolarization of the plasma membrane of nerve terminals in the control specimen was shown to significantly increase the MEPC frequency (quantal transmitter release) and exocytotic rate (FM 1-43 unloading from the synaptic vesicles preliminarily stained with the dye), which was caused by a rise in the intracellular Ca^2+^ concentration due to opening of voltage-gated Ca channels. A slight increase in the MEPC frequency and in the rate of synaptic vesicle exocytosis was observed under depolarization in case of blockade of Ca channels and chelating of intracellular Ca^2+^ ions (cooperative action of Cd^2+^ and EGTA-AM). The processes of synaptic vesicle endocytosis (FM 1-43 loading) were proportional to the number of synaptic vesicles that had undergone exocytosis both in the control and in case of cooperative action of Cd^2+^ and EGTA-AM. A hypothesis has been put forward that Ca-independent synaptic vesicle exo- and endocytosis that can be induced directly by depolarization of the membrane exists in the frog motor terminal in addition to the conventional Ca-dependent process.

## INTRODUCTION

Trasmitter release via synaptic vesicle exocytosis is the main function of presynaptic nerve terminals in a chemical synapse. Exocytosis is accompanied by processes of endocytosis (i.e., by the formation of new vesicles that are filled with the neurotransmitter and can participate in the transmitter release again) [1, 2]. It is believed that the exo- and endocytotic processes are induced under natural conditions due to an increase in the intracellular Ca^2+^ concentration as the voltage-gated Ca channels in the plasma membrane open [3-5].

Ca-dependence of the voltage-gated action of synaptic vesicle exocytosis is associated with specialized proteins, synaptotagmins I, II, IX, which are the main candidates as calcium ion sensors [6]. Spontaneous (asynchronous) exocytosis is also Ca^2+^-dependent and is determined by the action of intracellular Ca^2+^ on synaptotagmin I and Doc2b [7, 8]. The effect of calcium ions on endocytosis is more complex [9, 10]. An increase in the intracellular Ca2+ concentration can either induce/ accelerate endocytosis [11] or inhibit it [3, 9]. Calcium ion regulation of endocytosis can be mediated by calcineurin, Ca^2+^/calmodulin-dependent phosphatase, and calcium binding to synaptotagmin [12, 13].

However, there is a hypothesis that transmitter release can be controlled directly by changes in the membrane voltage of the nerve terminal without entry of Ca^2+^ [14, 15]. In ganglionic neurons, depolarization enhances exocytosis in a Ca-independent manner [16], while the subsequent endocytosis is independent of an increase in the intracellular Ca^2+^ concentration and shows a rapid dynamics [17].

The role of depolarization in transmitter release and synaptic vesicle exo- and endocytosis in a motor nerve terminal was studied in this work by electrophysiological and fluorescent methods.

## EXPERIMENTAL

**Study object, solutions**

Isolated nerve and muscle preparation from the cutaneous pectoris muscle of the frog *Rana ridibunda *in the winter season (December through February) were used for the experiments. The frogs were refrigerated at 5°C and transferred to the laboratory 2 h before the experiment. The work was carried out in compliance with international guidelines for the proper conduct of animal experiments.

The standard Ringer’s solution (115.0 mM NaCl, 2.5 mM KCl, 1.8 mM CaCl2, 2.4 mM NaHCO_3_) was used; a pH of 7.2–7.4 and temperature of 200°C were maintained. All the experiments were conducted only for the nerve terminals on the surface. In order to block the nerve terminal action potential, 1 μM tetrodotoxin was added to the perfusion solution. In some cases, Ringer’s solution supplemented with Cd^2+^ ions (0.2 mM) was used for blockage of the Ca^2+^ channels of the nerve terminal. To ensure binding of intracellular Ca^2+^ ions, the preparation was treated with the membrane-permeable form of EGTA calcium chelator (EGTA-AM) (50 μM) for 1 h. All the reagents used were purchased from Sigma (USA). The experiments were conducted at a constant perfusion rate of 5 ml/min; bath volume was 10 ml.

**Electrophysiology**

Miniature end plate currents (MEPC) were recorded using extracellular glass microelectrodes filled with a 2 M NaCl solution (~1 μm tip end; resistance of 1–5 MΩ). The electrode was applied to a nerve terminal at a distance of 20–40 μm from the final myelin segment. The signals were amplified using an extracellular amplifier and digitized using L-CARD 1250. The MEPC frequency was determined from the average time between two successive signals (impulses/s).

**Fluorescent microscopy**

A 6 μM FM1-43 fluorescent dye (SynaptoGreen C4, Invitrogen, USA) was used for the experiments. The marker was bound reversibly to the presynaptic membrane and became trapped inside the newly formed synaptic vesicles during endocytosis (was “loaded” into a nerve terminal) [18]. Fluorescence images were obtained using an Orca II CC D video camera (Hamamatsu, Japan) and an Olympus BX51 motorized microscope (Germany, Cell^P software) equipped with the DSU confocal system and an Olympus LUMPLFL 60xw lens. Terminal fluorescence in the central and distal portions of the nerve terminal was analyzed. The ImagePro program was used to assess the fluorescence intensity as relative fluorescence units of a pixel minus the background fluorescence. The background fluorescence was determined as the mean fluorescence intensity in a 50 X 50 pixel square in an image area showing no nerve terminal [19].

**Depolarization of the nerve terminal**

Two glass micropipettes with a 2–5 μm tip diameter filled with a 2 M NaCl solution were used to depolarize the nerve terminal. One (depolarizing) pipette was applied to the preterminal portion of the nerve terminal under visual control, while the second one was applied to the muscle fiber containing the nerve terminal at a distance of 1 mm from the first pipette. The stimulating pipettes were connected to a DS3 stimulator (Digitimer Ltd.) that was used as a current source. The current in the circuit was controlled with a microamperometer.

The statistical analysis was performed using the Origin Pro software. The quantitative results are presented as a mean ± standard error, *n *is the number of independent runs. Statistical significance was determined using the Student’s t- and ANOVA tests.

## RESULTS

**Electrophysiology. Transmitter release under depolarization of nerve terminals**

**Fig. 1:**
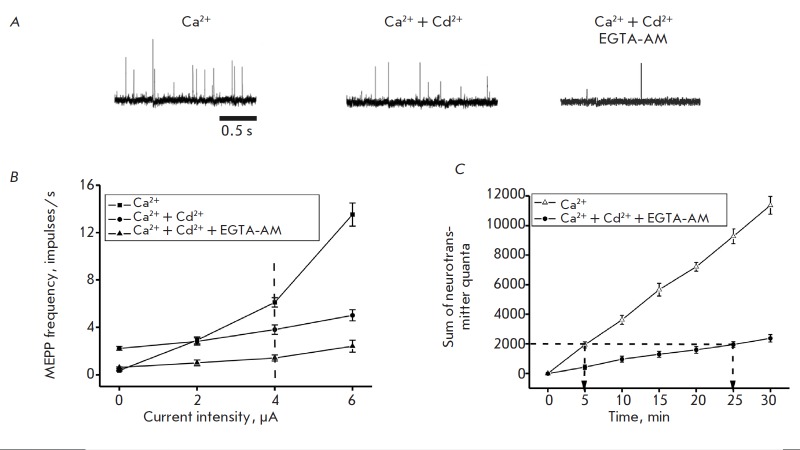
Effect of the depolarizing current on neurotransmitter release. *A *— MEPC frequency during the action of the depolarizing current (4 μA) in the control; Cd^2+^ ions were added, both EGTA-AM and Cd^2+^ were used. *B *— MEPC frequency as a function of the intensity of the depolarizing current. The dashed line indicates the MEPC frequency under a depolarizing current of 4 μA. *C *— Cumulative curves of transmitter release during the action of a depolarizing current of 4 μA. Y axis shows the sum of neurotransmitter quanta, X axis shows the time elapsed since the beginning of depolarization, min. The dashed line indicates the coordinates of the points corresponding to an identical sum of neurotransmitter quanta

At an extracellular Ca^2+^ concentration of 1.8 mM, the MEPC frequency was 0.23 ± 0.03 impulses/s (*n *= 25). Constant current depolarization of the membrane resulted in a rapid increase in the MEPC frequency (Fig. 1A), which was retained during the entire time that the current was applied (up to 40–50 min). The increase in the MEPC frequency depended on the current (Fig. 1B). Thus, the MEPC frequency increased to 2.9 ± 0.3 impulses/s (*n *= 10, *p * < 0.01) under a direct current (2 μA), while increasing to 6.1 ± 0.4 (*n *= 10,* p * < 0.01) and 12.9 ± 0.5 impulses/s (*n *= 10, *p * < 0.01) at 4 and 6 μA, respectively (Fig. 1 A, B).

Supplementation of the perfusion solution with Cd^2+^ ions (0.2 mM) increased the MEPC frequency to 2.22 ± 0.04 impulses/s (*n *= 20, *p * < 0.01). A weaker effect of depolarization on the MEPC frequency was observed in this case (Fig. 1 A, B). Thus, when a depolarizing current (2, 4 and 6 μA) was applied, the MEPC frequency reached 2.8 ± 0.3 (*n *= 10, *p * < 0.05), 3.8 ± 0.4 (*n *= 10,* p * < 0.01), and 5.2 ± 0.4 (*n *= 10, *p * < 0.01) impulses/s, respectively (Fig. 1 A, B).

An hour-long exposure to EGTA-AM caused no significant changes in the MEPC frequency, which was 0.20 ± 0.03 impulses/s (*n *= 16, *p *> 0.05) in this case. The preliminary treatment of the nerve-muscle preparation with EGTA-AM (see the Experimental section) eliminated the stimulating effect of Cd2+ ions (0.2 mM) on the MEPC frequency (Fig. 1 A, B). The MEPC frequency under these conditions (0.21 ± 0.02 impulses/s (*n *= 20, *p *> 0.05)) was identical to that for the control specimens. However, the stimulating effect of depolarization on the MEPC frequency was still observed, although it was weaker than that in the control or against the action of Cd^2+^ (Fig. 1 B). A depolarizing current of 2, 4, and 6 μA increased the MEPC frequency to 0.9 ± 0.2 (*n *= 10, *p * < 0.05), 1.5 ± 0.2 (*n *= 10, *p * < 0.01), and 2.8 ± 0.3 (*n *= 10, *p * < 0.01) impulses/s, respectively.

The rate and time dependence of transmitter secretion under constant current depolarization of the nerve terminal was analyzed using cumulative curves (Fig. 1 C). In this case, the sum of all the MEPC that had emerged vs. polarization time was plotted. Figure 1B shows the cumulative curves of transmitter release under depolarization of the nerve terminal (current of 4 μA) for 30 min. The number of quanta of neurotransmitter released from the nerve terminals in the control after a 5-min depolarization is equal to that released after a 25-min depolarization of a nerve terminal treated with EGTA-AM and in the presence of Cd^2+^ ions in an ambient environment (Fig. 1 C).

**Fluorescent microscopy. Depolarization of the nerve terminal and processes of synaptic vesicle endocytosis**

**Fig. 2:**
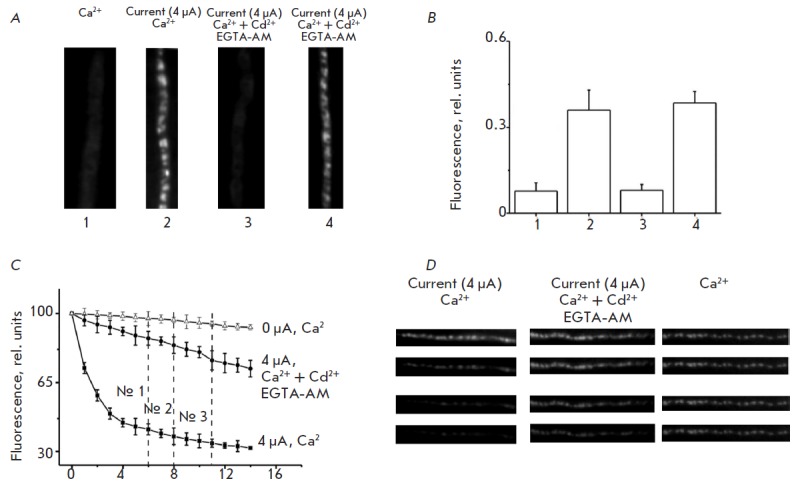
Synaptic vesicle exo- and endocytosis induced by depolarization of the nerve terminal membrane. *A *— Images of FM 1-43 fluorescence in the nerve terminal after application of FM 1-43 (25 min) at rest (*1*), under the depolarizing current for 5 min (*2*), and use of both EGTA-AM and Cd^2+^ during 5 (*3*) and 25 (*4*) min. *B *— Fluorescence intensity of the nerve terminals preliminarily stained with FM 1-43 according to different protocols: 1, 2, 3, 4. Y axis shows the fluorescence intensity (rel. units). *C *— Average fluorescent de-staining profiles during depolarization in the control (4 μA, Ca^2+^) and when using EGTA-AM and Cd^2+^ (4 μA, Ca^2+^+Cd^2+^+ EGTA-AM). The curve (0 μA, Ca^2+^) representing the changes in the fluorescence intensity at rest without depolarization is shown on the graph. Y axis shows fluorescent intensity, % (100% — fluorescent intensity before depolarization), X axis shows the time elapsed since the beginning of depolarization, min. *D *— Images of the FM 1-43 fluorescence of the nerve terminal at the times 0, №1, №2, and №3. The dashed lines (№1, №2, №3) designate fluorescence levels corresponding to times of 6, 8, and 11 min

The incubation of a nerve–muscle preparation in the standard Ringer’s solution with FM1-43 (5–40 min) caused nonspecific fluorescence of the nerve terminal (Fig. 2A) due to dye binding to the membrane [18-20]. The mean fluorescence intensity of the nerve terminal was 0.075 ± 0.005 rel. units (*n *= 32) (Fig. 2B). Intensely fluorescent spots along the nerve terminal could be seen after constant current depolarization (4 μA) of the nerve terminal for 5 min in the standard Ringer’s solution with FM1-43. These spots are an aggregation of vesicles that had undergone the exocytosis– endocytosis cycle and entrapped the fluorescent dye (Fig. 2A). In this case, the mean fluorescence intensity was 0.16 ± 0.01 rel. units (*n *= 27, *p * < 0.05) (Fig. 2B). When EGTA-AM and Cd^2+^ exerted a joint effect in addition to constant current depolarization of the membrane (4 μA) for 5 min, the dye was not loaded into the nerve terminal (nonspecific fluorescence of nerve terminal 0.08 ± 0.004 rel. units, *n *= 30, *p *>0.01) (Fig. 2 A, B). However, a longer constant current exposure (25 min) gave rise to fluorescent spots along the nerve terminal (0.17 ± 0.01 rel. units, *n *= 25, *p * < 0.05), attesting to the fact that endocytosis was occurring (Fig. 1 A,B).

**Dynamics of synaptic vesicle exocytosis under depolarization of nerve terminals**

In order to assess the synaptic vesicle exocytosis, we analyzed the dynamics of the decrease in the fluorescence intensity of nerve terminals that had been pre-loaded with a marker [18-20]. First, FM1-43 was loaded under a depolarization current (4 μA) for 5 min. After a rest period (1 h), a depolarization current (4 μA) was applied on the stained nerve terminals again, resulting in the release of the dye (through exocytosis) from synaptic vesicles and in a decrease in the fluorescence intensity of nerve terminals (Fig. 2 C, D). It should be mentioned that the fluorescent spots were observed in the standard Ringer’s solution for a long time (Fig. 2 C, D). An appreciably rapid and sharp decrease in the fluorescence of the preliminarily loaded nerve terminals was observed under constant current depolarization (4 μA) (Fig. 2 C, D). By the time the depolarization current had been applied for 2 min, the fluorescence intensity had fallen to 58 ± 3% (*n *= 10, *p * < 0.01), while 12–15 min later it became as low as ~30% of the initial level. If the preparations were treated with EGTA-AM prior to the loading of the dye and the nerve terminal membrane was subsequently subjected to constant current depolarization in the presence of Cd^2+^, the fluorescence intensity of a nerve terminal (unloading) occurred much slower (Fig. 2 C, D). Thus, the fluorescence intensity dropped to 95 ± 2% (*n *= 10, *p * < 0.01) after depolarization for 2 min, while the fluorescence intensity of the spots 12–15 min after remained at the level of ~70% of the initial one.

## DISCUSSION

In most studies focused on exo- and endocytosis, depolarization of the membrane was induced using a solution with an increased content of potassium ions [1, 20, 21]. However, the use of the solution changes the equilibrium potential for K^+^ and all the processes associated with the transport of K^+^ ions (e.g., function of Na/KATPase) and can also inhibit synaptic vesicle endocytosis [22]. Constant-current depolarization of the nerve terminal membrane, which does not have the side effects described above, was used in this study to assess the role of the membrane potential in synaptic vesicle exo- and endocytosis.

**Ca-independent exocytosis**

The experiments have demonstrated that constant current depolarization of the nerve terminal membrane at an extracellular concentration of calcium ions of 1.8 mM results in an increase of quantal transmitter release (MEPC frequency) and an appreciably rapid and wellpronounced unloading of FM1-43 (Fig. 1 B, Fig. 2 C). All these facts attest to the fact that depolarization of the nerve terminal membrane induces synaptic vesicle exocytosis due to the opening of the potential-gated Ca^2+^ channels, entry of Ca^2+^ ions into the nerve terminals, and activation of the fusion mechanism [1, 6, 23].

The next task was to assess the Ca^2+^ion values in depolarization- induced synaptic vesicle exocytosis. One could attempt to stimulate exocytosis in a calcium-free medium by depolarization; however, the removal of extracellular Ca^2+^ is fraught with the disturbance of the architecture of exocytic sites, the phase state of the membrane, the structure of membrane proteins and blocks synaptic vesicle endocytosis [10, 24]. Hence, all the experiments were conducted at a normal extracellular concentration of Ca^2+^ ions.

Cd^2+^ ions at a concentration of 0.2 mM are efficient and universal blockers of voltage-dependent Ca^2+^ channels of all (L-, N-, P/Q-, R-, and T-) types [25]. It has been demonstrated in experiments using Cd^2+^ that depolarization increases the MEPC frequency, although this rise is not as significant as that in the control (Fig. 1 B). It is an interesting fact that Cd^2+^ ions increase transmitter release to a certain extent (Fig. 1 B), which is also typical of other bi- and trivalent cations [27]. Cd^2+^ can affect the Ca^2+^-sensitive metabotropic receptor, whose activation induces the phospholipase C signaling pathway. Diacylglycerol (stimulating protein kinase C and exocytosis protein Munc13) and inositol trisphosphate (increasing the intracellular concentration of Ca^2+^ due to the release from the endoplasmic reticulum) are eventually formed in the nerve terminal [28]. It can be assumed that cadmium ions penetrate into a nerve terminal and cause an increase in the cytosolic calcium level due to its release from the calcium depot [29].

We have previously demonstrated that two buffers binding the intracellular Ca^2+^ ions –EGTAAM and BAPTA-AM (1,2-bis(2-aminophenoxy) ethane-N,N,N’,N’-ethylenediamine tetraacetic acid tetra(acetoxymethyl ester)) – suppress the increase in the MEPC frequency induced by a hyperpotassium solution (suppress the increase in the MEPC frequency induced by a solution with an increased content of potassium ions) to an identical extent, thus attesting to similar efficiencies in the chelating of the cytosolic Ca^2+^ [30]. EGTA-AM was used to eliminate the aforementioned effect of Cd^2+^. Indeed, there was no stimulating effect of Cd^2+^ ions on the MEPC frequency against chelating of intracellular Ca^2+^ (Fig. 1 B). Meanwhile, in the presence of EGTA-AM and blockage of Ca^2+^ entry into the extracellular environment, the depolarizing current caused a slight (but statistically significant) increase in the MEPC frequency (Fig. 1 B). The efficiency of a direct current (4 μA) in inducing synaptic vesicle exocytosis was also detected under these conditions by fluorescent microscopy, which could be observed as reduced fluorescence of the preliminarily loaded nerve terminals (Fig. 2 C). All these observations indicate that in addition to the conventional Ca^2+^-dependent exocytosis, an extracellular Ca^2+^-independent synaptic vesicle exocytosis also exists. This type of exocytosis is presumably induced by membrane depolarization under presynaptic voltage and is a component of the induced transmitter release.

**Ca-independent endocytosis**

Exo- and endocytosis processes are tightly coupled and occur at a 1:1 ratio; thus, the endocytosis intensity should be assessed only for an identical exocytosis intensity. According to the resulting data, we found that the number of quanta released from a nerve terminal in the control under depolarization for 5 min and current intensity of 4 μA is equal to that released during depolarization of the nerve terminal preliminarily treated with EGTA-AM for 30 min with Cd^2+^ ions added to the medium (Fig. 1 C). These findings were also confirmed by the results of experiments using a FM1-43 endocytic marker. Fluorescent spots of intensity almost identical to those in the control emerged in the nerve terminals under these conditions (Fig. 2 A, B). A hypothesis can be put forward that compensatory endocytosis can be induced both by an increase in the intracellular Ca^2+^ concentration when the voltage-gated calcium channels of the plasma membrane open [12, 13] and directly via depolarization of the nerve terminal membrane.

## CONCLUSION

The revealed dependence of exo- and endocytosis on the membrane voltage of a nerve terminal provides some additional potentialities for regulating transmitter release and synaptic transmission. No molecular targets for a direct effect of depolarization on synaptic vesicle exo- and endocytosis have been identified yet. However, recent studies have revealed the dependence on voltage in a large number of signaling molecules (protein kinases A and C, phosphatase of phosphoinositides conjugated to presynaptic autoreceptor G-proteins) affecting the mechanism of synaptic vesicle exo- and endocytosis [17, 31-33]. It is also possible that Ca^2+^ channels of the plasmatic membrane, which can transduce the depolarization signal to the SNARE complex and endocytosis proteins, are sensors that detect changes in the membrane voltage [14, 34].

## Abbreviations

EGTA-AM: ethylene glycol-O, O’-bis(2-aminoethyl)-N, N, N’, N’-tetraacetic acid acetoxymethyl ester
MEPC: miniature end plate currents
